# Endothelial cell tetrahydrobiopterin deficiency attenuates LPS-induced vascular dysfunction and hypotension^☆^

**DOI:** 10.1016/j.vph.2015.08.009

**Published:** 2016-02

**Authors:** Surawee Chuaiphichai, Anna Starr, Manasi Nandi, Keith M. Channon, Eileen McNeill

**Affiliations:** a British Heart Foundation Centre of Research Excellence, Division of Cardiovascular Medicine, Radcliffe Department of Medicine, University of Oxford, UK; b Wellcome Trust Centre for Human Genetics, University of Oxford, UK; c Pharmacology and Therapeutics Group, Institute of Pharmaceutical Science, Faculty of Life Sciences & Medicine, King's College London, UK

**Keywords:** *Gch1*, GTP cyclohydrolase 1, BH4, tetrahydrobiopterin, LPS, lipopolysaccharide, Tetrahydrobiopterin, Endothelial cell, LPS, Vascular dysfunction, Hypotension

## Abstract

Overproduction of nitric oxide (NO) is thought to be a key mediator of the vascular dysfunction and severe hypotension in patients with endotoxaemia and septic shock. The contribution of NO produced directly in the vasculature by endothelial cells to the hypotension seen in these conditions, *vs.* the broader systemic increase in NO, is unclear. To determine the specific role of endothelium derived NO in lipopolysaccharide (LPS)-induced vascular dysfunction we administered LPS to mice deficient in endothelial cell tetrahydrobiopterin (BH4), the essential co-factor for NO production by NOS enzymes. Mice deficient in endothelial BH4 production, through loss of the essential biosynthesis enzyme *Gch1* (*Gch1*^*fl*/*fl*^Tie2cre mice) received a 24 hour challenge with LPS or saline control. *In vivo* LPS treatment increased vascular GTP cyclohydrolase and BH4 levels in aortas, lungs and hearts, but this increase was significantly attenuated in *Gch1^fl/fl^*Tie2cre mice, which were also partially protected from the LPS-induced hypotension. In isometric tension studies, *in vivo* LPS treatment reduced the vasoconstriction response and impaired endothelium-dependent and independent vasodilatations in mesenteric arteries from wild-type mice, but not in *Gch1^fl/fl^*Tie2cre mesenteric arteries. *Ex vivo* LPS treatment decreased vasoconstriction response to phenylephrine in aortic rings from wild-type and not in *Gch1^fl/fl^*Tie2cre mice, even in the context of significant eNOS and iNOS upregulation. These data provide direct evidence that endothelial cell NO has a significant contribution to LPS-induced vascular dysfunction and hypotension and may provide a novel therapeutic target for the treatment of systemic inflammation and patients with septic shock.

## Graphical abstract

**Figure:**
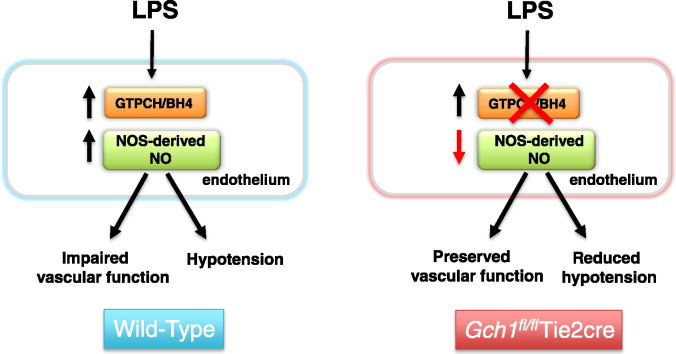

## 1 Introduction

Endotoxaemia is a leading cause of morbidity and mortality, characterised by systemic inflammation, decreased peripheral vascular resistance, microvascular leak and decreased cardiac output leading to refractory hypotension [24]. High levels of nitric oxide (NO) production by inducible nitric oxide synthase (iNOS, encoded by *NOS2*), which can be induced by lipopolysaccharide (LPS), are believed to be a key mediator of these phenomena [21]. The synthesis of NO by all NOS isoforms requires the cofactor tetrahydrobiopterin, BH4. Biosynthesis of BH4 is catalysed by GTP cyclohydrolase I (GTPCH), a rate limiting enzyme for *de novo* BH4 biosynthesis, which is encoded by *Gch1*. Increased circulating plasma biopterins and nitrite/nitrate have been reported in both animals and patients with septic shock [3], [10], [14], [15], [29], [31].

We have previously shown that *Gch1* expression is a key determinant of BH4 bioavailability, NOS regulation and thus NO generation in the vasculature of healthy mice [7], [9], [30]. In the vascular system, pro-inflammatory stimuli have been shown to increase the synthesis of BH4 levels by up-regulating *Gch1* mRNA and expression, that accompanies up-regulation of iNOS mRNA and protein in the endothelium and vascular smooth muscle [16], [22], [23]. Increased vascular iNOS-derived NO generation reduces vasocontractile response and causes hypotension which underlies pathophysiology of endotoxaemia and septic shock. The relevant contribution of endothelial NOS production to vascular dysregulation following systemic endotoxin exposure is unknown. Previous works have also demonstrated the important role of endothelial NOS (eNOS) in the pathogenesis of LPS-induced endotoxaemia and septic shock that eNOS activity is the key determinant of iNOS expression and activity in murine model of septic shock [8], [32]. Indeed, mice with global eNOS deficiency are protected against LPS-induced vascular dysfunction and hypotension due to loss of iNOS expression and activity [8], [32].

Systemic treatment of mice with a non-selective GTPCH inhibitor, 2,4-diamino-6-hydroxypyrimidine (DAHP) reduces BH4 levels, vascular NOS-derived NO generation and reduces a degree of hypotension in an experimental model of septic shock, despite no change in induction of iNOS [3], [26], suggesting a role for *Gch1* and BH4 biosynthesis in the pathogenesis of septic shock. Furthermore, mice with global iNOS deficiency are protected against LPS-induced vascular dysfunction and hypotension [20], [32]. However, systemic administration of non-selective NOS inhibitors has been shown to have inconsistent effects in both experimental models and patients with septic shock [2], [13], [18]. These observations highlight the need to better understand the mechanistic role of the NOS enzymes in different cell types in the pathophysiology of endotoxaemia and septic shock. It is not clear whether endothelial cell-specific *vs.* systemic effects of NOS are important.

We have utilised a mouse model with endothelial cell-specific deletion of BH4 biosynthesis to investigate the importance of endothelial cell-derived NO production in the vascular and hemodynamic responses to LPS-induced endotoxaemia.

## 2 Material and methods

### 2.1 Animals

All animal studies were conducted with ethical approval from the Local Ethical Review Committee and in accordance with the UK Home Office regulations (Guidance on the Operation of Animals, Scientific Procedures Act, 1986). Mice were housed in ventilated cages with a 12-hour light/dark cycle and controlled temperature (20–22 °C), and fed normal chow and water ad libitum.

### 2.2 *Gch1* conditional endothelial knockout mice

We have generated a *Gch1* conditional knockout (floxed) allele using Cre/loxP strategy. Exons 2 and 3 of *Gch1*, encoding for the active site of GTPCH I, were flanked by two loxP sites in a targeting construct that was used to produce *Gch1^fl/fl^* mice after homologous recombination in embryonic stem cells. Pups carrying the *Gch1* floxed allele were then back-crossed for 8 generations to the C57Bl/6J line. Once back-crossed the resultant *Gch1^fl/fl^* animals were bred with Tie2cre transgenic mice to produce *Gch1^fl/fl^*Tie2cre mice where *Gch1* is deleted in endothelial cells, generating a novel mouse model of endothelial cell-specific BH4 deficiency mouse [7]. The Tie2cre transgene is active in the female germline, as such only male animals are used to establish breeding pairs to maintain conditional expression. Mice were genotyped according to the published protocol [7].

### 2.3 Non-invasive blood pressure measurement using tail-cuff method

Blood pressure in conscious wild-type and *Gch1^fl/fl^*Tie2cre mice was measured using the Visitech BP-2000 tail-cuff plethysmography system. Experiments were performed between the hours of 8:00 am and 12.00 pm. Twenty readings were taken per mouse of which the first 5 readings were discarded. The remaining 15 readings were used to calculate the mean systolic blood pressure and heart rate in each mouse. Following 5 days of training, basal blood pressure and heart rate were recorded for 3 consecutive days.

### 2.4 Administration of LPS

LPS (1 mg/kg body weight; Sigma-Aldrich, Gillingham, UK) or sterile saline was administered in male wild-type and *Gch1^fl/fl^*Tie2cre mice (16–22 week-old) via intraperitoneal (i.p.) route. Mice were injected between 8:00 and 9:00 am for all studies to avoid diurnal variation in the response to LPS. Mice were monitored throughout the study for adverse effects. LPS administration is expected to cause a systemic inflammation and hypotension, which may lead to hypothermia. To counteract this, mice were maintained in a heated recovery cage and were given a subcutaneous injection of saline in accordance with local ethical requirements. 24-hour post injection, mice were culled and tissues were collected. The dose of LPS used was not lethal in any experimental animals.

### 2.5 Determination of tissue tetrahydrobiopterin levels

BH4 and oxidised biopterins (BH2 and biopterin) were determined by high-performance liquid chromatography (HPLC) followed by electrochemical and fluorescence detection, respectively, following an established protocol [4]. Briefly, either a small piece of tissue (approximately 20 mg) or a whole aorta was resuspended in ice-cold resuspension buffer (50 mM phosphate-buffered saline, 1 mM dithioerythritol, 1 mM EDTA, pH 7.4), and either homogenised (for tissues) or subjected to three freeze–thaw cycles (for aortas). After centrifugation at 13,200 rpm for 10 min at 4 °C, supernatant was removed and ice-cold acid precipitation buffer (1 M phosphoric acid, 2 M trichloroacetic acid, 1 mM dithioerythritol) was added. Following centrifugation at 13,200 rpm for 10 min at 4 °C, the supernatant was removed and injected onto the HPLC system. Quantification of BH4 and oxidised biopterins was obtained by comparison with external standards and normalised to protein concentration, determined by the BCA protein assay.

### 2.6 Isometric tension vasomotor studies

Vasomotor function was analysed using isometric tension studies in a wire myograph (Multi-Myograph 610M, Danish Myo Technology, Denmark). Briefly, mice were culled by overdose of inhaled isofluraneand vascular rings were isolated from the thoracic aorta or mesenteric arcades. The aortic rings or 2nd mesenteric arteries (2 mm) were mounted on a wire myograph containing 5 ml of Krebs–Henseleit buffer (KHB [in mmol/l]: NaCl 120, KCl 4.7, MgSO_4_ 1.2, KH_2_PO_4_ 1.2, CaCl_2_ 2.5, NaHCO_3_ 25, glucose 5.5) at 37 °C, gassed with 95% O_2_/5% CO_2_. After allowing vessels to equilibrate for 30 min, the optimal tension was set (equivalent to 100 mm Hg). The vessel viability was tested using 60 mM KCl. Concentration–response contraction curves were established using cumulative half-log concentrations of phenylephrine and U46619 in the presence or absence of 100 μM of non-selective NOS inhibitor, L-NAME. Acetylcholine was used to stimulate endothelium-dependent vasodilatations in increasing cumulative concentrations. Responses were expressed as a percentage of the pre-constricted tension. The NO donor sodium nitroprusside (SNP) was used to test endothelium-independent smooth muscle relaxation in the presence of 100 μM L-NAME. All pharmacological drugs were pre-incubated at least 20 min before the dose–response curves were determined. All drugs used were purchased from Sigma Chemical Company.

### 2.7 NOx determination

Lung homogenates and plasma were deproteinated in acid precipitation buffer (1 M phosphoric acid, 2 M trichloroacetic acid), and the nitrite and nitrate content was quantified using a CLD88 NO analyser (Ecophysics).

### 2.8 Western blot analysis

Western blots were performed with anti- GTPCH (a gift from S. Gross, Cornell University New York), anti- iNOS (Abcam) and anti- eNOS (BD Bioscience) antibodies in vascular tissues from wild-type and *Gch1^fl/fl^*Tie2cre mice, using standard protocols.

### 2.9 Quantification real-time RT-PCR

RNA were reserve transcribed using Superscript II (Life Technologies) according to standard protocols. 5 ng RNA equivalent cDNA was used to perform real-time PCR using pre-designed TaqMan gene expression assays (Life Technologies) using a BioRad CFX1000. Gene expression levels of mouse *Gch1*, *Nos3* and *Nos2* were normalised to the housekeeping gene *GAPDH* using the Delta Ct method.

### 2.10 Statistical analysis

Data are expressed as mean ± standard error of the means and analysed using GraphPad Prism version 5.0 (San Diego, USA). Comparisons between WT and *Gch1^fl/fl^*Tie2cre were made by unpaired *Student's* t test. Concentration–response curves were compared by two-way analysis of variance for repeated measurements followed by the Bonferroni *post-hoc* test. A *P*-value of less than 0.05 was considered statistically significant.

## 3 Results

### 3.1 Endothelial cell-targeted *Gch1* deletion and BH4 deficiency attenuates lipopolysaccharide-induced hypotension

**Fig. 1:**
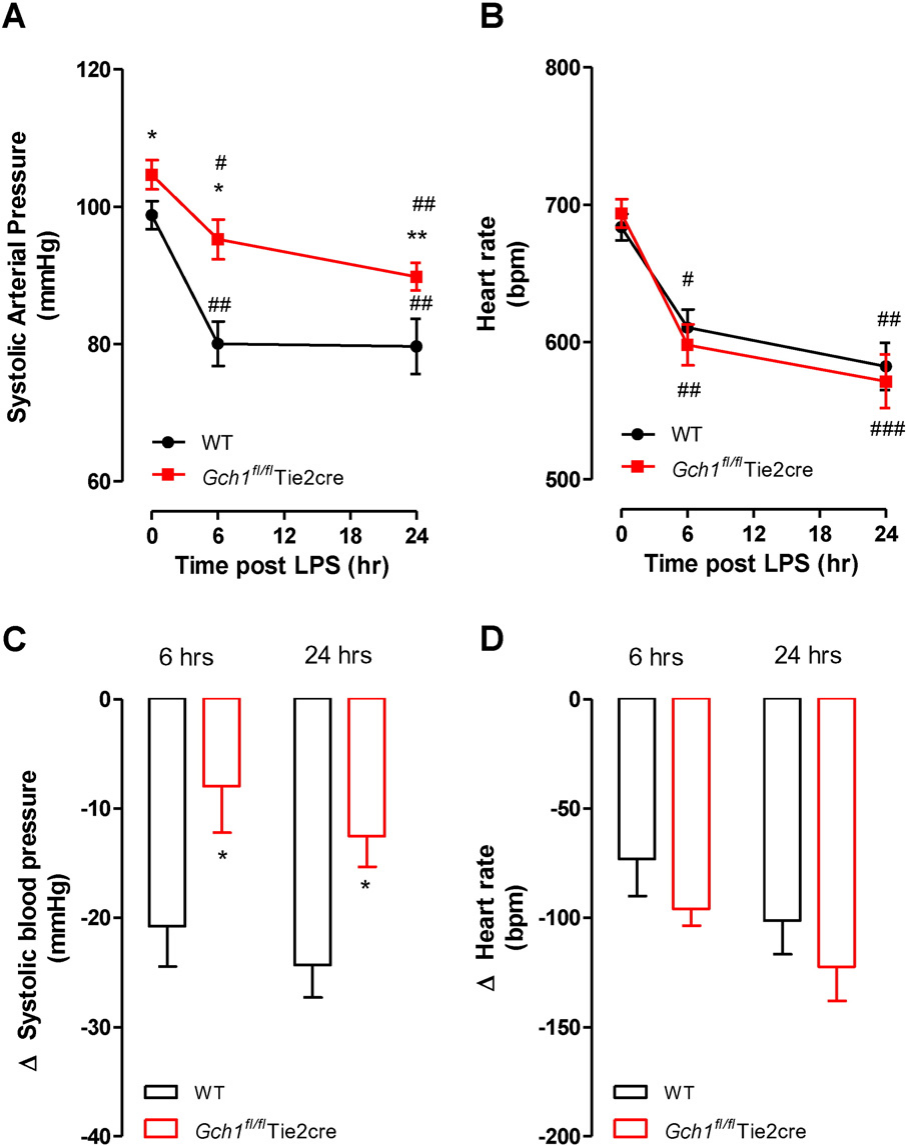
Effect of LPS on hemodynamic parameters in *Gch1^fl/fl^*Tie2cre mice. Mice from both genotypes either received a single dose of 1 mg/kg lipopolysaccharide (LPS) or saline control i.p. and then underwent non-invasive tail-cuff recordings. A) Systolic blood pressure (mm Hg), and B) heart rate (beat per minute) were monitored at baseline, 6 and 24 h following injection. The C) change in blood pressure and D) heart rate from baseline at the two timepoints was calculated (*P < 0.05, **P < 0.01 comparing genotypes, *#P* < 0.05, *##P* < 0.01 comparing treatment; n = 6 animals per group).

We generated matched litters of *Gch1^fl/fl^*Tie2cre and *Gch1^fl/fl^* mice by crossing male *Gch1^fl/fl^*Tie2cre with female *Gch1^fl/fl^* mice (hereafter referred as wild-type). Body weights between the groups were similar. Blood pressure recordings were performed at 6 h and 24 h post LPS administration (1 mg/kg i.p.). As was the case with our previous study [7], baseline systolic blood pressure was significantly increased in *Gch1^fl/fl^*Tie2cre mice compared to wild-type littermates (105.8 ± 2.2 mm Hg *versus* 98.8 ± 2.0 mm Hg; *P* < 0.05). Six-hours after LPS injection, systolic blood pressures were significantly decreased in both wild-type (80.1 ± 3.2 mm Hg; *P* < 0.01) and *Gch1^fl/fl^*Tie2cre mice (95.3 ± 2.9 mm Hg; *P* < 0.05) (Fig. 1A), but with a significantly greater reduction in blood pressure in wild-type mice compared to *Gch1^fl/fl^*Tie2cre mice (change in blood pressure, − 20.8 ± 3.7 mm Hg in wild-type *versus* − 7.9 ± 4.2 mm Hg in *Gch1^fl/fl^*Tie2cre mice; *P* < 0.05) (Fig. 1C). 24 h after LPS injection, *Gch1^fl/fl^*Tie2cre mice remained significantly less hypotensive than wild-type mice (change in BP from baseline, − 24.3 ± 3.0 mm Hg in wild-type *versus* − 12.5 ± 2.8 mm Hg in *Gch1^fl/fl^*Tie2cre mice; *P* < 0.05). Baseline heart rate was similar between wild-type and *Gch1^fl/fl^*Tie2cre mice. Administration of LPS significantly reduced the heart rate of both genotypes at 6 h after injection (from 680 ± 12 bpm to 603 ± 17 bpm in wild-type mice; from 694 ± 10 bpm to 598 ± 15 bpm in *Gch1^fl/fl^*Tie2cre mice) and 24-hour post injection (592 ± 22 bpm in wild-type mice; 572 ± 20 bpm in *Gch1^fl/fl^*Tie2cre mice) (Fig. 1B and D), but this was not significantly different between genotypes. As expected, saline treatment had no significant effect on systolic blood pressures or heart rates in either genotype (data not shown).

### 3.2 Increased vascular GTPCH and BH4 levels are attenuated in *Gch1^fl/fl^*Tie2cre mice following LPS in vivo

**Fig. 2:**
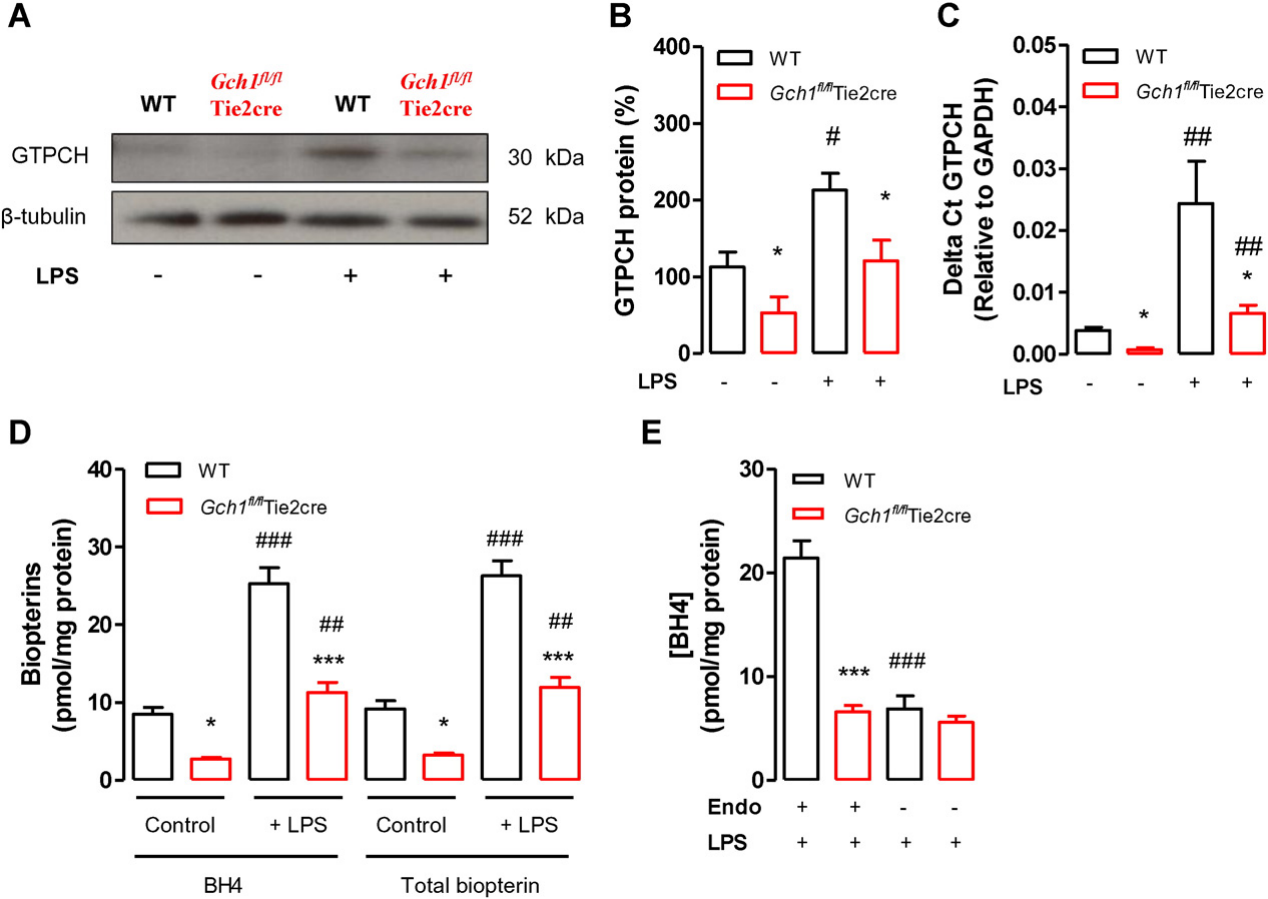
Vascular GTPCH and BH4 levels in aortas. A) Representative immunoblots showing aortic GTP cyclohydrolase (GTPCH) protein in wild-type and *Gch1^fl/fl^*Tie2cre mice either treated with saline control or 1 mg/kg lipopolysaccharide (LPS) for 24 h, with corresponding quantitative data in B), measured as percentage band density of β-tubulin (a loading control) (*P < 0.05; comparing genotypes, #*P* < 0.05; comparing treatment; n = 6 aortas per group). C) Quantitative real-time PCR was used to quantify *Gch1* gene expression in aortic extracts (*P < 0.05; comparing genotypes, ##*P* < 0.01; comparing treatment; n = 4 aortas per group). D) Aortic BH4 and total biopterin levels, measured by HPLC, were significantly reduced in aortas from saline-treated *Gch1^fl/fl^*Tie2cre mice compared to saline-treated wild-type littermates. Following 1 mg/kg LPS treatment for 24 h, aortic BH4 and total biopterin levels were significantly elevated in both genotypes, but the difference still maintained (*P < 0.05; ***P < 0.001 comparing genotypes, ##*P* < 0.01, ###*P* < 0.001 comparing treatment; n = 6 to 8 aortas per group). E) Vascular BH4 in endothelium-denuded aortas from wild-type and *Gch1^fl/fl^*Tie2cre mice following LPS *in vivo* (***P < 0.001 comparing genotypes, ###*P* < 0.001 comparing treatment; n = 4 to 5 aortas per group).

Gene expression and western blot analysis of aortic extracts confirmed that basal vascular *Gch1* expression and GTPCH protein were significantly reduced in aortas from saline-treated *Gch1^fl/fl^*Tie2cre mice when compared with saline-treated wild-type controls (Fig. 2A, B, and C). Following LPS treatment, vascular *Gch1* expression and GTPCH protein were increased in both wild-type and *Gch1^fl/fl^*Tie2cre mice, but remained significantly higher in wild-type mice (Fig. 2A, B, and C). This was accompanied by a significant decrease in vascular BH4 and total biopterin levels in saline-treated *Gch1^fl/fl^*Tie2cre mice compared to saline-treated wild-type mice (*P* < 0.05) (Fig. 2D). LPS treatment also increased vascular BH4 and total biopterin levels. However, the increase in vascular BH4 following LPS was significantly attenuated when endothelial BH4 production was absent in keeping with GTPCH protein expression (*P* < 0.01) (Fig. 2D). Furthermore, vascular BH4 levels were significantly decreased by ≈ 70% in endothelial-denuded aortas from wild-type mice following LPS treatment such that vascular BH4 was no longer different between LPS treated wild-type and LPS treated *Gch1^fl/fl^*Tie2cre mice. Removal of the endothelium has no significant effect on vascular BH4 levels in LPS treated *Gch1^fl/fl^*Tie2cre mice, suggesting a complete excision of *Gch1* gene with Tie2 in endothelial cells in this model. This finding indicates significant upregulation of vascular GTPCH protein and thus BH4 biosynthesis in the endothelium following LPS *in vivo* (Fig. 2E).

### 3.3 LPS treatment has no effect on vascular reactivity in aortas in both wild-type and endothelial cell BH4 deficient mice

**Fig. 3:**
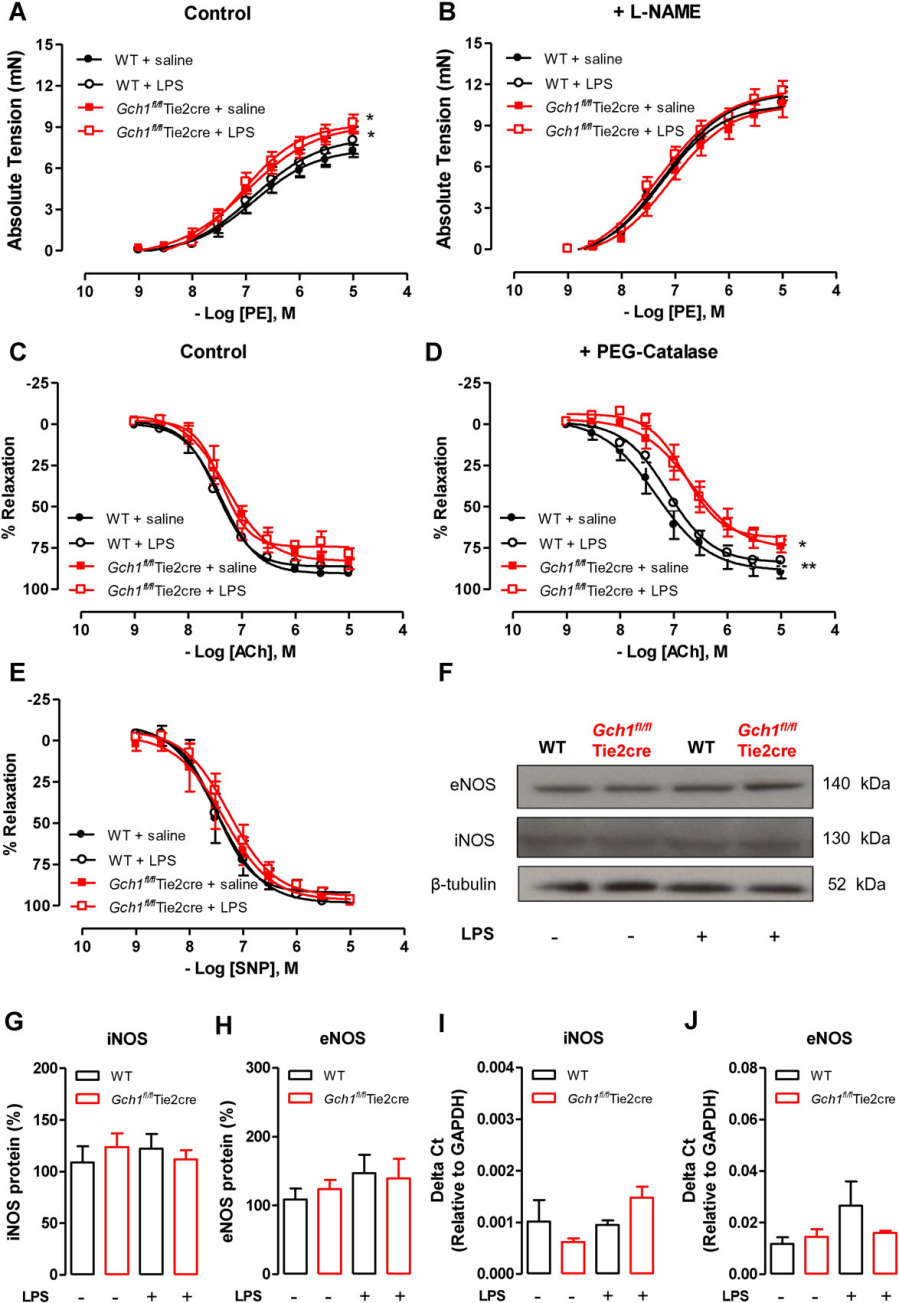
Effect of LPS on vascular reactivity in isolated aortas *in vivo.* Vasoconstriction in response to phenylephrine A) alone or B) in the presence of non-selective nitric oxide synthase inhibitor, L-NAME (100 mM) in aortic rings from wild-type and *Gch1^fl/fl^*Tie2cre either treated with saline control or 1 mg/kg lipopolysaccharide (LPS) for 24 h (*P < 0.05; comparing genotype; n = 6 to 8 animals per group). Endothelium-dependent vasodilatation to acetylcholine C) alone or D) in the presence of catalase-polyethylene glycol (PEG-catalase; 400 units/ml) (*P < 0.05, **P < 0.01; comparing genotype; n = 4 to 6 animals per group). E) Endothelium-independent vasodilatation to sodium nitroprusside (SNP). F) Representative immunoblots showing endothelial nitric oxide synthase (eNOS) and inducible NOS (iNOS) protein in aortas from wild-type and *Gch1^fl/fl^*Tie2cre treated with either saline control or LPS *in vivo* with corresponding quantitative data in G and H), measured as percentage band density of β-tubulin (a loading control) (n = 6 aortas per group). I and J) Quantitative real-time PCR was used to quantify iNOS and eNOS gene expression in aortic extracts from wild-type and *Gch1^fl/fl^*Tie2cre either treated with saline control or LPS *in vivo* (n = 4 aortas per group).

We next determined the effect of LPS on vascular reactivity in conduit vessels. As was the case with our previous study [7], we found that vasoconstriction in response to phenylephrine was significantly enhanced in *Gch1^fl/fl^*Tie2cre aortas compared to wild-type controls (*P* < 0.05). This difference was normalised in the presence of L-NAME (Fig. 3A and B). Endothelium-dependent vasodilatation was minimally impaired in *Gch1^fl/fl^*Tie2cre aortas compared to wild-type controls (Fig. 3C). In the presence of H_2_O_2_ scavenger, catalase-polyethylene glycol (PEG-catalase), endothelium-dependent vasodilatations were significantly inhibited in *Gch1^fl/fl^*Tie2cre aortas but unchanged in wild-type aortas (Fig. 3D). There was no difference in endothelium-independent vasodilatation to SNP between the genotypes (Fig. 3E).

Despite an increase in aortic GTPCH and BH4 levels following *in vivo* LPS treatment, aortic vasoconstriction and vasodilatation were unaffected by LPS treatment (Fig. 3A, B, C, D and E). Gene expression and western blot analysis demonstrated that LPS treatment had no significant effect on either aortic eNOS or iNOS expression or protein in either wild-type or *Gch1^fl/fl^*Tie2cre mice (Fig. 3F, G, H, I and J). This finding suggests that upregulation of vascular GTPCH and BH4 levels alone, without alteration of NOS expression, has no significant effect on vasomotor function in conduit vessels following LPS treatment *in vivo*.

### 3.4 Increased vascular GTPCH, BH4 levels, and NO generation are attenuated in tissues from *Gch1^fl/fl^*Tie2cre mice following LPS in vivo

**Fig. 4:**
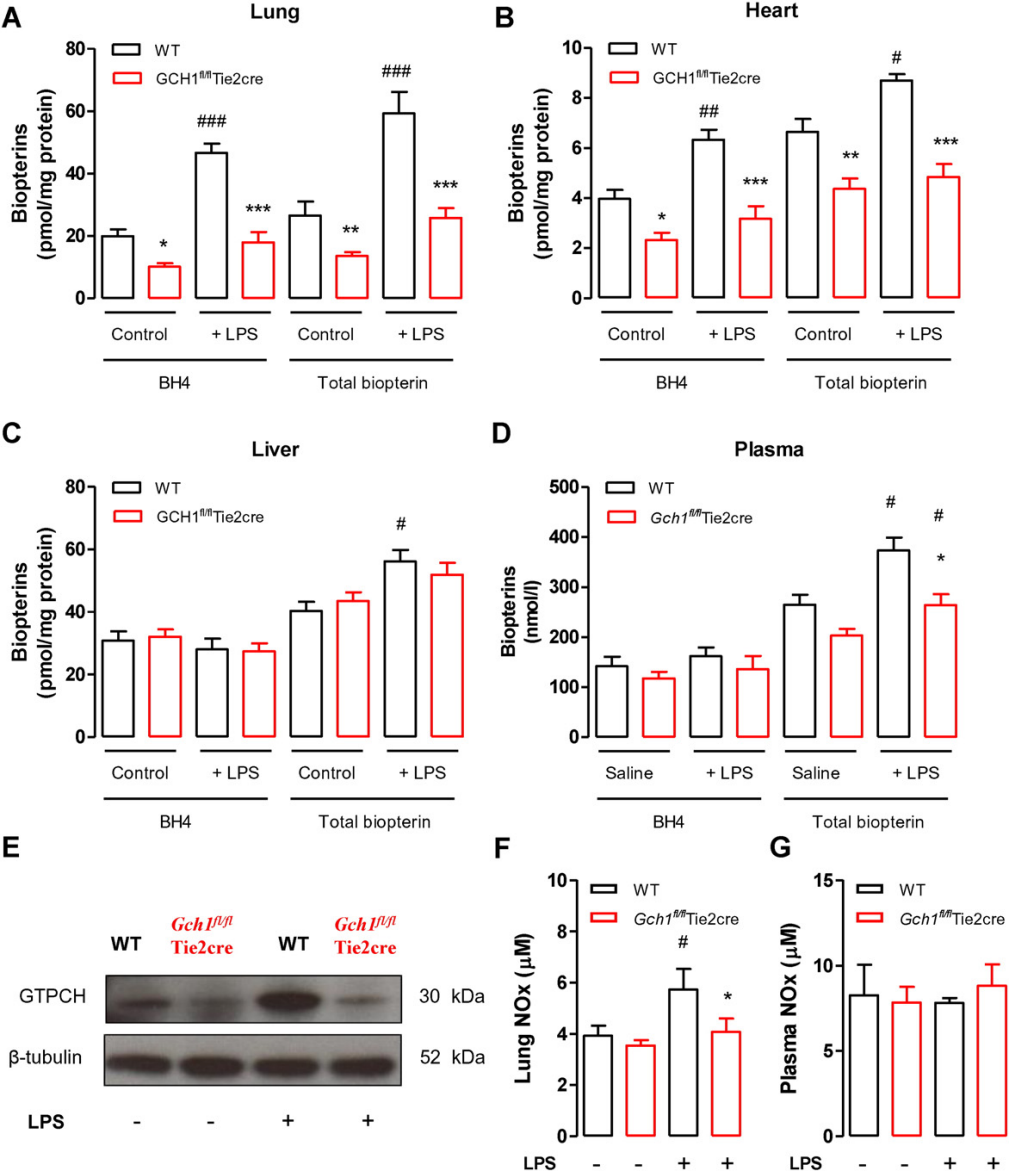
BH4 levels, GTPCH protein, and NO production in endotoxaemic mice. The levels of BH4 and total biopterin, measured by HPLC, in A) lung, B) heart, C) liver, and D) plasma from wild-type and *Gch1^fl/fl^*Tie2cre either treated with saline control or 1 mg/kg LPS for 24 h (*P < 0.05; **P < 0.01; ***P < 0.001 comparing genotypes, #*P* < 0.05, ##*P* < 0.01, ###*P* < 0.001 comparing treatment; n = 4 to 6 animals per group). E) Representative immunoblots showing GTP cyclohydrolase (GTPCH) protein in lung homogenates from wild-type and *Gch1^fl/fl^*Tie2cre either treated with saline control or LPS. Total nitrite and nitrate content, measured by NO analyser, in F) lung homogenates and G) plasma following saline control or LPS treatment *in vivo* (*P < 0.05; comparing genotypes, #*P* < 0.05, comparing treatment; n = 4 to 6 animals per group).

To determine whether the dose of LPS given was sufficient to cause systemic alteration of *Gch1* and NOS biology we analysed further endothelial cell-rich tissues, such as lung and heart. As previously observed BH4 and total biopterin levels were significantly reduced in saline treated *Gch1^fl/fl^*Tie2cre mice when compared with saline treated wild-type mice (Figs. 4A and 3B). Following LPS treatment, BH4 and total biopterin levels were significantly increased in both lung and heart tissues from wild-type mice and slightly increased but not statistically significant in *Gch1^fl/fl^*Tie2cre mice (Fig. 4A and B). However this regulation of BH4 levels by LPS treatment was not seen in all tissues, as the liver showed no significant difference in BH4 levels between saline treated mice and LPS treated mice in either wild-type or *Gch1^fl/fl^*Tie2cre mice (Fig. 4C). However, dihydrobiopterin (BH2), which lacks NOS cofactor activity, levels were significantly increased in the liver from both wild-type and *Gch1^fl/fl^*Tie2cre mice following LPS, such that the BH4/(BH2 + biopterin) ratio was significantly reduced in both wild-type and *Gch1^fl/fl^*Tie2cre mice (Fig. S1). Furthermore, western blot analysis of lung extracts confirmed that LPS treatment caused a significant increase in GTPCH protein in wild-type but unchanged in *Gch1^fl/fl^*Tie2cre mice (Fig. 4E). This was accompanied by a significant increase in nitrite/nitrate content in lung homogenates from LPS treated wild-type mice, which was not detected in LPS treated *Gch1^fl/fl^*Tie2cre mice, indicating that NOS activity is altered in the *Gch1^fl/fl^*Tie2cre mice.

In plasma, there was no detectable difference in basal BH4 and total biopterin levels between saline-treated *GCH1^fl/fl^*Tie2cre and saline-treated wild-type mice (Fig. 4D). Following LPS treatment, plasma BH4 levels were unchanged, but plasma total biopterin levels were significantly increased in both wild-type and *Gch1^fl/fl^*Tie2cre mice, indicating an increase in oxidised biopterins (Fig. 4D). There was no significant difference in plasma nitrite/nitrate production between saline treated mice and LPS treated mice in either wild-type or *Gch1^fl/fl^*Tie2cre mice (Fig. 4G). These data indicated that the dose of LPS used, although sufficient to cause alteration in biopterin and NOS biology does not cause an overwhelming NOS activation, as may be observed with higher doses of LPS [10].

### 3.5 Endothelial cell BH4 deficiency reduces LPS-induced vascular dysfunction in resistance mesenteric arteries

**Fig. 5:**
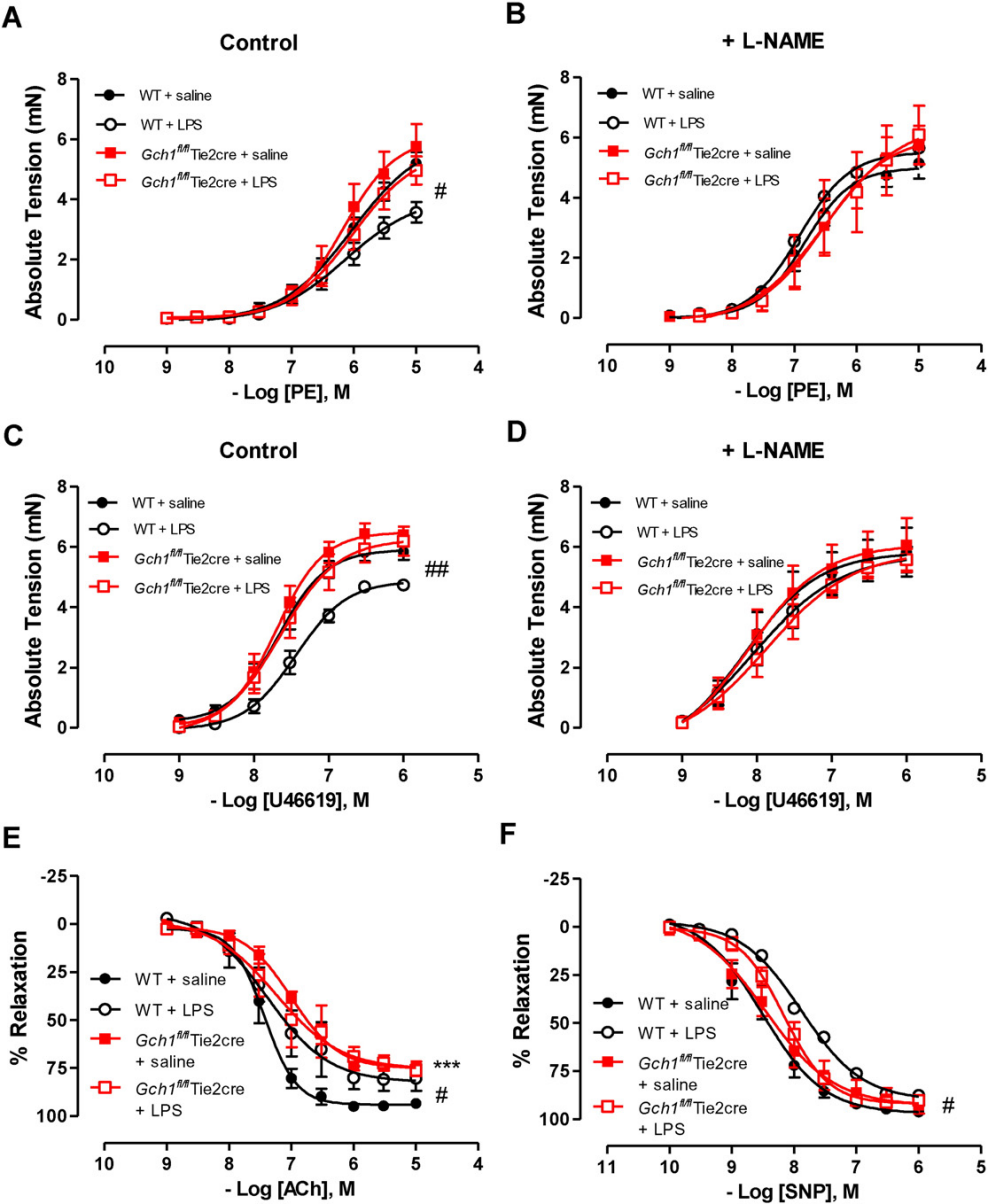
Effect of LPS on vascular reactivity in isolated mesenteric arteries. Vascular reactivity in 2nd mesenteric arteries was determined using wire myograph. Vasoconstriction in responses to A) phenylephrine (PE) and C) U46619 was attenuated in 2nd order mesenteric arteries from lipopolysaccharide (LPS)-treated wild-type mice compared to saline-treated wild-type mice. LPS treatment had no significant effect on vasoconstriction in responses to either PE or U46619 in *Gch1^fl/fl^*Tie2cre mesenteric arteries (#*P* < 0.05, ##*P* < 0.01; n = 8 to 10 animals per group). B and D) Vasoconstriction in responses to PE and U46619 were normalised in the presence of non-selective nitric oxide synthase inhibitor (100 mM; L-NAME). The effect of LPS on E) endothelium-dependent vasodilatation to acetylcholine (Ach) and F) endothelium-independent vasodilatation to sodium nitroprusside (SNP) (#*P* < 0.05, ***P < 0.001; n = 4 to 6 animals per group).

To investigate the relationships between blood pressure and changes in the resistance vasculature, mesenteric arteries from wild-type and *Gch1^fl/fl^*Tie2cre mice were harvested from LPS or saline-treated animals 24 h after injection. In wild-type mesenteric arteries, LPS administration significantly attenuated the contractile response to the α-adrenoceptor agonist phenylephrine (PE) (Fig. 5A; *P* < 0.05). This blunting of the contractile response was prevented in the presence of L-NAME (Fig. 5B), such that there was no longer a significant difference in vasoconstriction between saline-treated and LPS-treated wild-type mice. This indicates that increased NOS-derived NO is responsible for the decreased vasoconstrictor response in mesenteric arteries from LPS-treated wild-type. Similar findings were also observed when thromboxane A2 agonist U46619 was used (Fig. 5C; *P* < 0.01), suggesting that the blunted vasoconstrictor response was not due to specific alteration of receptor signalling on vascular smooth muscle cells. In contrast to these observations in wild-type mice, LPS treatment in *Gch1^fl/fl^*Tie2cre mice resulted in no significant alteration in vasoconstrictor response to either PE or U46619 (Fig. 5A and B respectively). Furthermore, L-NAME treatment had no effect on contractile response in LPS-treated *Gch1^fl/fl^*Tie2cre mice (Fig. 5B and D).

In saline treated mice, endothelium-dependent vasodilatation in response to acetylcholine was significantly impaired in *Gch1^fl/fl^*Tie2cre mesenteric arteries when compared with that in wild-type mesenteric arteries (*P* < 0.001). Following LPS treatment, endothelium-dependent vasodilatation was significantly impaired in wild-type mesenteric arteries (*P* < 0.05), but unaltered in *Gch1^fl/fl^*Tie2cre mesenteric arteries, such that endothelium-dependent vasodilatation was no longer different between LPS treated wild-type and LPS-treated *Gch1^fl/fl^*Tie2cre mesenteric arteries (Fig. 5E). Furthermore, LPS treatment significantly reduced the potency of endothelium-independent vasodilatation to SNP in wild-type mesenteric arteries when compared to mesenteric arteries from saline treated wild-type mice (Fig. 5F; *P* < 0.05). In contrast, LPS treatment has no significant effect on endothelium-independent vasodilatation to SNP in *Gch1^fl/fl^*Tie2cre mesenteric arteries. This finding indicates that endothelial cell BH4 regulates LPS-induced vascular dysfunction in resistance arteries.

### 3.6 Endothelial cell BH4 deficiency prevents LPS-induced aortic dysfunction *ex vivo*

**Fig. 6:**
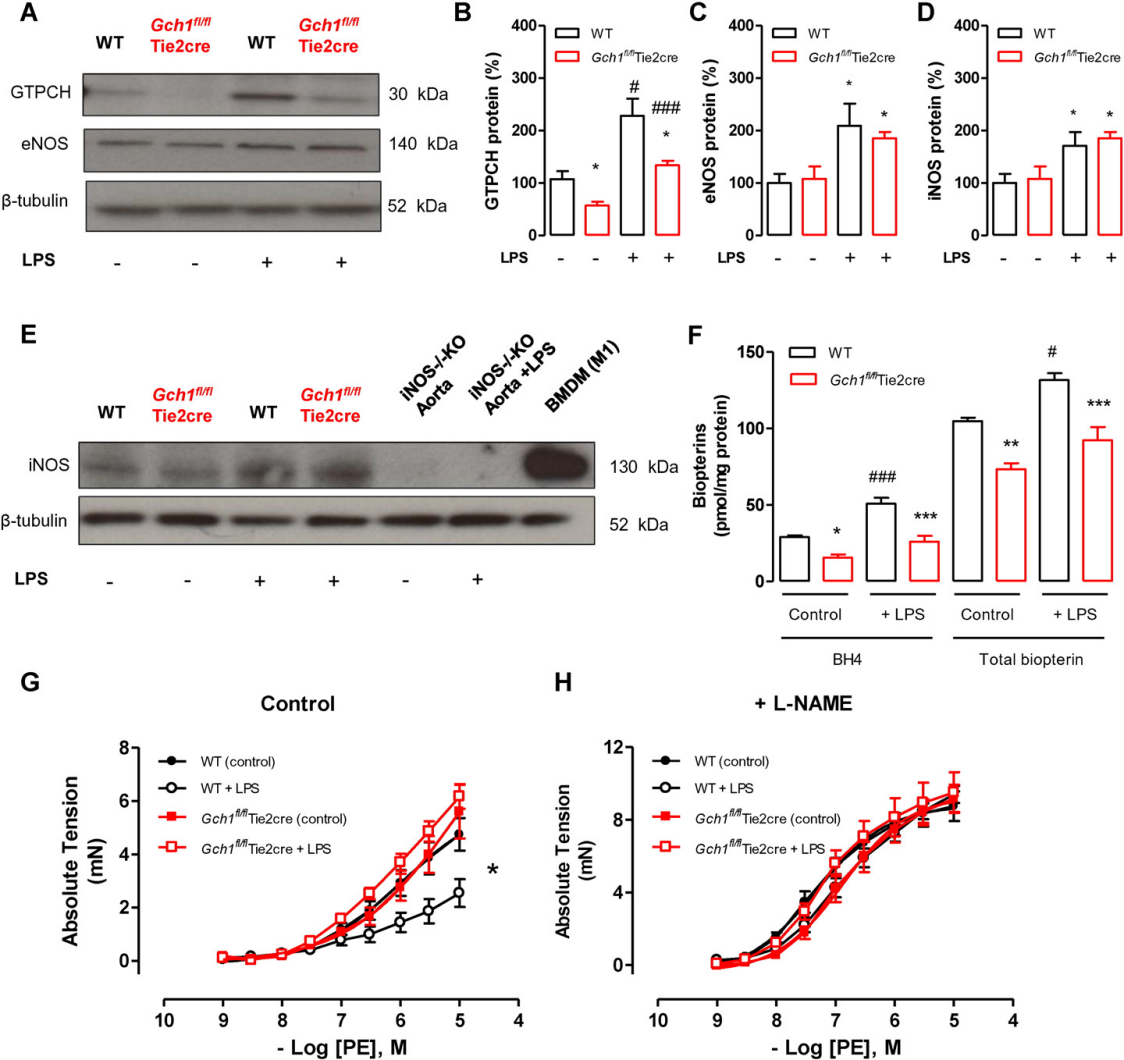
Effect of *ex vivo* LPS treatment on wild-type and *Gch1*^*fl/fl*^*Tie2cre* aortas. A) Representative immunoblots showing GTP cyclohydrolase (GTPCH), endothelial nitric oxide synthase (eNOS) protein in wild-type and *Gch1^fl/fl^*Tie2cre aortas incubated with either Dulbecco's Modified Eagle Medium (DMEM) alone or DMEM containing 1 μg/ml lipopolysaccharide (LPS) for 24 h at 37 °C. B) Quantitative data, measured as percentage band density of β-tubulin, showing GTPCH, eNOS and iNOS protein in wild-type and *GCH1^fl/fl^*Tie2cre aortas incubated with either saline control or LPS. C) Representative immunoblots with corresponding quantitative data below showing inducible NOS (iNOS) protein in wild-type and *Gch1^fl/fl^*Tie2cre aortas incubated with either saline control or LPS. The identity of the bands obtained was confirmed using control lysate from activated bone marrow derived macrophage and iNOS^−/−^ aortas, and iNOS^−/−^ aorta treated with LPS (*P < 0.05; n = 5 per groups). D) Tetrahydrobiopterin (BH4) and total biopterin levels, measured by HPLC, in wild-type and *Gch1^fl/fl^*Tie2cre aortas incubated with either saline control or LPS for 24 h. Vascular reactivity in aortic rings was determined using wire myograph. E) Vasoconstriction to phenylephrine (PE). F) Vasoconstriction to PE in the presence of non-selective nitric oxide synthase inhibitor (L-NAME) (*P < 0.05 WT control *vs.* WT + LPS, n = 5 to 6 aortas per group).

To determine the effect of endothelial cell BH4 deficiency on vascular function in response to a higher dose of LPS and a clear induction of vascular iNOS expression, isolated mouse aortic rings from wild-type and *Gch1^fl/fl^*Tie2cre mice were incubated in Dulbecco's Modified Eagle Medium (DMEM) with or without 1 μg/ml of LPS for 24 h. Following incubation, GTPCH protein expression, BH4 and total biopterin levels were significantly increased in both wild-type and *Gch1^fl/fl^*Tie2cre aortas (Fig. 6A, B and F). The induction of this pathway was significantly greater in wild-type aortas, indicating the endothelial component of this response. Western blot analysis demonstrated that eNOS and iNOS protein expressions were also significantly increased in both wild-type and *Gch1^fl/fl^*Tie2cre aortas following LPS incubation (Fig. 6A, C, D and E). Importantly, the induction of both eNOS and iNOS by LPS was of a similar magnitude in both genotypes.

We next investigated the effect of endothelial cell BH4 deficiency on vasomotor function in aortic rings treated with LPS *ex vivo*. Incubation with LPS significantly blunted vascular contractile function of wild-type aortic rings in response to phenylephrine (PE) compared to saline-treated wild-type controls. In the presence of L-NAME, vasoconstriction was increased such that vasoconstriction was no longer different between controls and LPS-treated aortas. In *Gch1^fl/fl^*Tie2cre aortas, incubation with LPS had no significant effect on vasocontractility compared to saline-treated *Gch1^fl/fl^*Tie2cre aortas (Fig. 6G and H).

## 4 Discussion

In this study, we have demonstrated the specific role of endothelial cell BH4-dependent NOS regulation in the pathogenesis of LPS-induced vascular dysfunction and hypotension. The major findings of this study are as follows. First, LPS treatment results in a significant increase in vascular GTPCH and BH4 levels in wild-type mice, but the magnitude of this increase is attenuated in *Gch1^fl/fl^*Tie2cre mice, demonstrating a specific endothelial cell component of this response. Second, *in vivo* LPS treatment causes a reduction in vasoconstrictor responses and an impairment of endothelium-dependent and independent vasodilatation in mesenteric arteries from wild-type mice, which are preserved in mesenteric arteries from *Gch1^fl/fl^*Tie2cre mice. Third, *ex vivo* LPS treatment causes a NOS-mediated reduction in vasoconstrictor responses in wild-type aortas, which again does not occur in *Gch1^fl/fl^*Tie2cre aortas despite induction of iNOS and eNOS protein expression in both genotypes. Fourth, the lack of endothelial cell BH4 results in an attenuation of LPS-induced hypotension. Together these findings demonstrate for the first time that deficiency in endothelial cell *Gch1* and thus BH4 biosynthesis is alone sufficient to protect against LPS-induced vascular dysfunction and hypotension induced by the dose of LPS used here, indicating a novel role of endothelial cell *Gch1* and BH4-dependent NOS regulation in the pathogenesis of LPS-induced vascular dysfunction and hypotension.

A large body of evidence has demonstrated that administration of bacterial LPS causes an increase in vascular *Gch1* mRNA, GTPCH protein expression and BH4 levels in a coordinated manner with iNOS mRNA such that vascular iNOS-derived NO is increased [3], [21], [26]. Consistent with this, we found that *in vivo* LPS treatment causes an increase in vascular GTPCH protein and BH4 levels in endothelial cell-rich tissues such as aortas, lungs and hearts from wild-type mice. We have previously reported that the majority of vascular GTPCH and BH4 biosynthesis (~ 70%) are contributed by the endothelium in healthy mice [7] and human [11]. Thus, the endothelium may be a principal site of BH4 synthesis following LPS treatment. Indeed, endothelial-denudation leads to a reduction of vascular BH4 levels in aortas from LPS treated wild-type but unaltered in LPS treated *Gch1^fl/fl^*Tie2cre mice, demonstrating involvement of endothelial cells in GTPCH and BH4 biosynthesis in LPS-induced endotoxaemia. However, as a significant induction of BH4 is still observed in *Gch1^fl/fl^*Tie2cre aortic tissues, other cell types must also upregulate BH4 production.

When a higher dose of LPS was applied to aortic rings *ex vivo*, an increase in aortic iNOS protein in both wild-type and *Gch1^fl/fl^*Tie2cre mice was observed. Interestingly, aortic eNOS expression was also markedly increased in both genotypes following LPS *ex vivo*. This increased eNOS expression may act as a protective mechanism in endotoxaemia contributing to the maintenance of microcirculatory flow. However, as the *ex vivo* incubation system occurs in the absence of blood flow and the resulting sheer stress on the vessel wall care must be taken in extrapolating these results to the *in vivo* situation. However, increased eNOS expression has been documented in mesenteric arteries and skeletal muscle from an *in vivo* experimental model of LPS-induced endotoxaemia [1], [6]. In contrast, eNOS expression is decreased in vascular tissues from experimental models of severe septic shock [5], [12], indicating that vascular eNOS expression is dependent on the severity of disease induced by LPS. In the present study we found that the reduced vasoconstrictor response is reversed by L-NAME in mesenteric arteries from wild-type mice following LPS *in vivo*, indicating that this is mediated by LPS-induced increases in tonic NOS-derived NO. In contrast to wild-type mesenteric arteries, LPS treatment has no effect on vasoconstrictor responses in mesenteric arteries from *Gch1^fl/fl^*Tie2cre mice. Similar findings were also observed in conduit arteries (aortas) from *Gch1^fl/fl^*Tie2cre mice pre-incubated with LPS *ex vivo*.

Interestingly, endothelium-dependent and independent vasodilatations were impaired in mesenteric arteries from wild-type mice following LPS *in vivo*. This finding was consistent with previous reports, which had also described a decrease in sensitivity to NO following LPS treatment, and this was shown to be iNOS dependent in the mesentery [5], [19]. In contrast, LPS had no significant effect on either endothelium-dependent or independent vasodilatation in *Gch1^fl/fl^*Tie2cre mice following LPS *in vivo*, indicating that the inability to induce endothelial cell *Gch1* expression, BH4 biosynthesis and thus NOS-derived NO generation is likely to be the mechanism underlying the blunted decrease in vasoconstrictor response and preserved endothelium-dependent and independent vasodilatation in *Gch1^fl/fl^*Tie2cre mice. Previous works have demonstrated that eNOS activity is the key determinant of iNOS expression and activity in murine model of septic shock [8], [32]. Consistent with the previous reports, it is possible that deficiency in endothelial cell BH4 reduces eNOS activity and thus iNOS expression and activity, due to a loss of iNOS expression and/or BH4-dependent iNOS activity in *Gch1^fl/fl^*Tie2cre mice following LPS treatment. In this study, we demonstrated for the first time that deficiency of BH4 in the endothelial cell alone is sufficient to protect from LPS-induced vascular dysfunction in mesenteric arteries *in vivo*.

Mice with global iNOS deficiency are protected against LPS-induced vascular dysfunction and hypotension [5], [20]. However, it is not clear whether endothelial cell-specific *vs.* systemic effects of NOS are important in the pathogenesis of LPS-induced endotoxaemia induced by high doses of LPS or sepsis. In this study, we demonstrated for the first time that deficiency of BH4 in endothelial cells alone is protective against LPS-induced hypotension. However, endotoxaemia and sepsis seen in critically ill patients and more severe animal models are typified by a more profound decrease in blood pressure that causes organ failure through underperfusion and death. Under these conditions iNOS is expressed widely both locally within other vessel wall cells such as smooth muscle cells, and in other non-vascular cells. In the data presented here the lack of endothelial cell *Gch1* and reduced BH4 levels, whilst providing significant protection, is not sufficient to entirely prevent hypotension. The drop in blood pressure that is still observed in *Gch1^fl/fl^*Tie2cre mice is likely to be induced by the overproduction of iNOS-derived NO from non-endothelial cell sources. Indeed, we found a significant increase in vascular BH4 levels in endothelium-denuded aortas from *Gch1^fl/fl^*Tie2cre mice following LPS *in vivo*, suggesting the contribution of vascular GTPCH and BH4 levels from non-endothelial cells in this model. Increased iNOS and *Gch1* expression in vascular smooth muscle cells has been reported to be associated with vascular dysfunction and hypotension induced by LPS [16], [23]. Consistent with this idea, GTPCH feedback regulatory protein (GFRP) binding to GTPCH is reported to cause allosteric negative feedback regulation in the presence of excess BH4 production. The GFRP over expressing mice demonstrate that limiting BH4 synthesis in smooth muscle cells is partially protective from hypotension in the caecal ligation and puncture (CLP) model of sepsis [28]. Interestingly, heart rate was significantly reduced in both wild-type and *Gch1^fl/fl^*Tie2cre mice following LPS treatment, indicating LPS mediated physiological changes that are independent of blood pressure. This finding is consistent with previous reports both at low dose and high dose of LPS [25], [27] where bradycardia following LPS has been reported to be associated with a down-regulation of β1-adrenoceptor level in the myocardium [27].

Although deficiency in endothelial cell *Gch1* and BH4 protects against LPS-induced vascular dysfunction and hypotension in *Gch1^fl/fl^*Tie2cre mice using this non-lethal LPS dosing regimen, it is unknown whether deficiency in endothelial cell *Gch1* and BH4 biosynthesis is protective against endotoxaemia caused by LPS at higher doses or during septic shock *in vivo*. Investigation using vascular smooth muscle cell-specific *Gch1* deletion (e.g. SM22-cre mice) mice or knockout of *Gch1* in both endothelial cells and vascular smooth muscle cells may provide an insight into understanding the role of *Gch1* and BH4-dependent NOS regulation in different vessel wall cells in the pathophysiology of LPS-induced endotoxaemia and septic shock.

Systemic administration of non-selective NOS inhibitors *N^G^*-methyl-l-arginine (L-NMMA) has been shown to reduce plasma nitrite and nitrate and increase systemic vascular resistance and blood pressure in humans with septic shock; it did not however improve mortality in patients with septic shock [2], [13], [18]. Similarly, inhibition of NOS has been shown to have inconsistent effect in experimental model of endotoxaemia and septic shock. These observations indicate that the role of NO in endotoxaemia and septic shock is complex. This is likely due to the opposing local effects in the microcirculation. Reduced local NO in organ microcirculation, due to NOS inhibitor treatment, may lead to microcirculation hypoperfusion and organ damage in the model of endotoxaemia and septic shock. In contrast, supplementation of BH4 has been shown to maintain microcirculation flow and perfusion and increase the rate of survival in ovine model of peritoneal sepsis [17]. Thus, achieving the right balance between reducing hypotension, whilst maintaining organ perfusion in microcirculation may have therapeutic potential in the treatment of septic patients and understanding the cell type and enzymatic source of the NO contributing to both pathologies may be key to achieving this.

## 5 Conclusions

We have demonstrated for the first time that deficiency in endothelial cell *Gch1* and BH4 biosynthesis protects against LPS-induced vascular dysfunction and hypotension. These findings suggest that endothelial cell BH4-dependent NOS regulation plays a critical role in the pathogenesis of this LPS-induced endotoxaemia. Thus, targeting endothelial cell *Gch1* and BH4 biosynthesis may provide a novel therapeutic target for the treatment of circulatory collapse in patients with septic shock.

The following are the supplementary data related to this article.

Supplementary Fig. 1 Effect of LPS on dihydrobiopterin (BH2) and BH4/(BH2 and biopterin) ratio.
LPS treatment leads to evidence of BH4 oxidation in the lung and liver. Wild-type and *Gch1^fl/fl^*Tie2cre mice were treated with either saline control or 1 mg/kg lipopolysaccharide (LPS) for 24 h. Lung, heart and liver were harvested and processed for biopterin analysis by HPLC. A) BH2 level was significantly increased in lung homogenates from wild-type mice but attenuated in *Gch1^fl/fl^*Tie2cre mice following LPS *in vivo*. B) There was a trend decrease but not significant in BH4/(BH2 and biopterin) ratio in lung homogenates from both wild-type and *Gch1^fl/fl^*Tie2cre mice following LPS *in vivo* (***P < 0.001 comparing genotype; ###*P* < 0.001, comparing treatment; n = 4 to 6 animals per group). C and D) LPS treatment had no significant effect on either BH2 level or BH4/(BH2 + biopterin) ratio in heart homogenates from either wild-type and *Gch1^fl/fl^*Tie2cre mice. E) BH2 level was significantly increased in liver homogenates from both genotypes following LPS *in vivo*, which resulted in F) a striking reduction in the BH4/(BH2 + biopterin) ratio in both genotypes (#*P* < 0.05 comparing treatment; n = 4 to 6 animals per group).

## Disclosures

None.
