# Supplementary material for: Endothelial cell tetrahydrobiopterin deficiency attenuates LPS-induced vascular dysfunction and hypotension
Source: Vascul Pharmacol. 2016 Feb;77:69–79. doi: 10.1016/j.vph.2015.08.009 (PMC4746318; doi:10.1016/j.vph.2015.08.009)
Supplement: Supplementary Fig. 1 — Effect of LPS on dihydrobiopterin (BH2) and BH4/(BH2 and biopterin) ratio. LPS treatment leads to evidence of BH4 oxidation in the lung and liver. Wild-type and Gch1fl/flTie2cre mice were treated with either saline control or 1 mg/kg lipopolysaccharide (LPS) for 24 h. Lung, heart and liver were harvested and processed for biopterin analysis by HPLC. A) BH2 level was significantly increased in lung homogenates from wild-type mice but attenuated in Gch1fl/flTie2cre mice following LPS in vivo. B) There was a trend decrease but not significant in BH4/(BH2 and biopterin) ratio in lung homogenates from both wild-type and Gch1fl/flTie2cre mice following LPS in vivo (***P < 0.001 comparing genotype; ###P < 0.001, comparing treatment; n = 4 to 6 animals per group). C and D) LPS treatment had no significant effect on either BH2 level or BH4/(BH2 + biopterin) ratio in heart homogenates from either wild-type and Gch1fl/flTie2cre mice. E) BH2 level was significantly increased in liver homogenates from both genotypes following LPS in vivo, which resulted in F) a striking reduction in the BH4/(BH2 + biopterin) ratio in both genotypes (#P < 0.05 comparing treatment; n = 4 to 6 animals per group). [file mmc1.doc]

**SUPPLEMENTAL MATERIAL for**

**Endothelial Cell Tetrahydrobiopterin Deficiency Attenuates LPS-Induced Vascular Dysfunction and Hypotension**

Surawee Chuaiphichai ^a,b^, Anna Starr ^c^, Manasi Nandi ^c^, Keith M. Channon ^a,b^ , Eileen McNeill ^a,b,c,*^

^a^ British Heart Foundation Centre of Research Excellence,

Division of Cardiovascular Medicine, Radcliffe Department of Medicine, University of Oxford

^b^ Wellcome Trust Centre for Human Genetics, University of Oxford

^c^ Pharmacology and Therapeutics Group, Institute of Pharmaceutical Science, Faculty of Life Sciences & Medicine, King’s College London

***Corresponding Author:**

Dr Eileen McNeill,

British Heart Foundation Centre of Research Excellence,

Division of Cardiovascular Medicine,

University of Oxford,

John Radcliffe Hospital,

Oxford, OX3 9DU, UK

Tel: +44-1865-287662

Email: [eileen.mcneill@well.ox.ac.uk](mailto:eileen.mcneill@well.ox.ac.uk)

**SUPPLEMENTAL FIGURES AND FIGURE LEGENDS**

**Supplementary Figure 1. Effect of LPS on dihydrobiopterin (BH2) and BH4/(BH2 and biopterin) ratio.**

LPS treatment leads to evidence of BH4 oxidation in lung and liver. Wild-type and *Gch1^fl/fl^*Tie2cre mice were treated with either saline control or 1 mg/kg lipopolysaccharide (LPS) for 24 hours. Lung, heart and liver were haervested and processed for biopterin analysis by HPLC. **A)** BH2 level was significantly increased in lung homogenates from wild-type mice but attenduated in *Gch1^fl/fl^*Tie2cre mice following LPS *in vivo*. **B)** There was a trend decrease but not significant in BH4/(BH2 and biopterin) ratio in lung homogenates from both wild-type and *Gch1^fl/fl^*Tie2cre mice following LPS *in vivo* (****P<0.001* comparing genotype; ### *P<0.001*, comparing treatment; n=4 to 6 animals per group). **C and D)** LPS treatment had no significant effect on either BH2 level or BH4/(BH2+biopterin) ratio in heart homogenates from either wild-type and *Gch1^fl/fl^*Tie2cre mice. **E)** BH2 level was significantly increased in liver homogenates from both genotypes following LPS *in vivo*, which resulted in **F)** a striking reduction in the BH4/(BH2+biopterin) ratio in both genotypes (#*P<0.05* comparing treatment; n=4 to 6 animals per group).
